# Novel insights on Nerve growth factor mediated regulation of immune response

**DOI:** 10.1002/alz70855_102734

**Published:** 2025-12-23

**Authors:** Ruchi Gera, Simone Tambaro, Per Nilsson, Sumonto Mitra, Maria Eriksdotter

**Affiliations:** ^1^ Division of Clinical Geriatrics, Dept.of NVS, Karolinska Institutet, Huddinge, Stockholm, Sweden; ^2^ Division of Neurogeriatrics, Dept of NVS, Karolinska Institutet, Solna, Stockholm, Sweden; ^3^ Theme Inflammation and Aging, Karolinska University Hospital, Huddinge, Stockholm, Sweden

## Abstract

**Background:**

Alzheimer's disease (AD) is an age‐associated dementia disorder without a cure with altered metabolism of the neurotrophin‐ nerve growth factor (NGF) in the brain. We previously reported that exogenous NGF therapy showed promise in restoring cognitive decline in AD patients. Recently, the role of the immune system in AD pathology is being actively investigated in relation to inflammation. Currently, our understanding of the NGF‐immune cell interaction is limited. This study intends to expand this avenue by exploring NGF receptors expression on immune cells.

**Method:**

Splenic immune cells isolated from C57BL/6 mice were examined for NGF receptor (Tropomyosin receptor kinase A: TrkA) expression by flowcytometry. In addition, splenocytes were stimulated with PMA‐ionomycin ex‐vivo and treated with mature NGF for 48hr. Following stimulation, cells were collected to examine the levels of pro‐inflammatory TNF‐a cytokine by flow cytometry.

**Result:**

Differential expression of the NGF receptor TrkA in immune cells from innate and adaptive immune system were evaluated, Among innate immune cells, we found that dendritic cells (DC) showed higher levels of TrkA compared to macrophage and NK cells. B and T cells from adaptive immunity possessed comparable expression of TrkA but lower than DC. Among the subsets of T cells i.e. CD4, CD8, follicular helper T (Tfh) cells and regulatory T (Treg) cells, Tfh cells presented more TrkA than CD4, CD8 and Treg cell. Also, central memory CD4 T cells expressed more TrkA than the effector memory and naïve CD4 T cells. We also checked their biological relevance in T cells at an ex‐vivo platform. We observed that exogenous NGF supplementation reduced TNF‐a cytokine production from stimulated splenic‐T cells.

**Conclusion:**

Our results show that all the immune cells which we examined express NGF receptor TrkA, thus possessing the ability to respond to NGF. Our findings suggest, that NGF‐TrkA signaling can modulate the immune response in T cells by reducing inflammatory cytokine production.

